# Effects of individualized follow-up with a smartphone-application after cardiac rehabilitation: protocol of a randomized controlled trial

**DOI:** 10.1186/s13102-019-0148-2

**Published:** 2019-11-21

**Authors:** Pernille Lunde, Asta Bye, Astrid Bergland, Birgitta Blakstad Nilsson

**Affiliations:** 1 Department of Physiotherapy, Faculty of Health Sciences, OsloMet – Oslo Metropolitan University, PB 4, St. Olavs plass, 0130 Oslo, Norway; 2 Department of Nursing and Health Promotion, Faculty of Health Sciences, OsloMet – Oslo Metropolitan University, Oslo, Norway; 3 Regional Advisory Unit for Palliative Care, Department of Oncology, Oslo University Hospital, Oslo, Norway; 4 Section for Physiotherapy, Division of Medicine, Oslo University Hospital, Oslo, Norway

**Keywords:** mHealth, Cardiac rehabilitation, Mobile phone app, Smartphone, Lifestyle

## Abstract

**Background:**

Cardiac rehabilitation (CR) programs are evidence-based and widely recommended. However, benefits from CR are likely lost among individuals who discontinue their regular exercise routines and healthy habits. One possible approach to enhance adherence to lifestyle advice after completion of CR, may be individualized follow-up enabled by a smartphone application (app).

**Methods:**

A protocol of a single-blinded, pragmatic randomized controlled trial. The study will take place in the eastern part of Norway, and will include heart patients who have completed CR. Participants will be recruited from two CR centers. Based on power calculation, 113 participants will be included. The intervention group will receive individualized follow-up through an app on a weekly basis throughout a year. The app will be set up with the participant’s own goals, and the follow-up will be based on these individual goals. The control group will receive usual care, including general advice regarding physical activity, exercise and diet. The participants will be assessed at baseline (at completion of CR) and 12 months after baseline. Primary outcome of the study will be peak oxygen uptake. Secondary outcomes include exercise performance, quality of life, health status, health literacy, self-perceived goal achievement, exercise habits, body weight, blood pressure as well as lipids and triglycerides.

**Discussion:**

To our knowledge, this will be the first study to examine the effects of individualized follow-up with an app for one year, in patients completing CR. Hence, it is reasonable to assume that the study may be groundbreaking. Due to the large sample size and the theoretically based intervention, the study has the potential to generate new knowledge that may improve the design of future technology-based follow-up interventions of patients that have completed rehabilitation.

**Trial registration:**

ClinicalTrials.gov. NCT03174106. First registration, 19/05/2017.

## Background

Beneficial effects of cardiac rehabilitation (CR) have been well demonstrated [1]. Exercise is recommended as a core component in CR, as exercise is an independent measure of cardiovascular (CV) risk, both in healthy individuals and in patients with coronary disease [1–4]. However, benefits from CR are likely lost among individuals who discontinue their regular exercise routines and healthy habits. Participants in CR are often insufficiently prepared for independent exercise in their home environment and the beneficial effects of CR tends to decrease after completed CR [5]. One possible approach is to use mobile health interventions, such as smartphone-applications (apps), as a follow-up tool to promote adherence to lifestyle advice.

The benefit of an app is that it offers an opportunity for health-staff to give feedback to patients directly [6–9]. The use of apps for cardiac patients is shown to be promising regarding risk factor control, completion of CR, delivery of CR and adherence to the app [9–11]. Further, authors have pointed out apps as potential interventions for adherence to lifestyle advice in cardiac patients after completing CR [9, 12]. In that respect, apps may meet the need for long-term support, highlighted as necessary in the latest European guidelines on cardiovascular disease prevention in clinical practice for behavior change [3]. To our knowledge, no other studies have previously evaluated individualized follow-up with an app for one year after completing CR.

Our research group performed an experimental, pre-post single arm trial, lasting for 12 weeks to assess the feasibility of using an app for promoting and monitoring patients adherence to a heart-healthy lifestyle after CR [13]. Fourteen patients were recruited during spring 2017. The evaluation of feasibility was assessed through recruitment rate, adherence to the app including satisfaction with the technology, resource requirements and efficacy regarding capability to detect a change in quality of life (QoL) and health status questionnaires, and in perceived goal achievement. Results showed that 71% of patients who completed CR were eligible for the study. Further, all included participants (*n* = 14) used the app for preventive activities throughout the study period. Satisfaction with the technology was high, and all included participants found the technology-based follow-up both useful and motivational. Despite this, some potential improvements of the intervention were discovered [13]. Therefore, guidance from the participants in the feasibility study has been taken along and has been adopted and incorporated into the final design of the randomized controlled trial (RCT) which will be presented in this paper.

The primary aim of the planned study is to evaluate the effects of individualized follow-up, enabled through an app, on exercise capacity as determined by direct measurement of peak oxygen uptake (VO_2peak_), compared to standard care, one year after completion of CR. The second aim is to evaluate the effect of the intervention on exercise performance, QoL, health status, health literacy, self-perceived goal achievement, exercise habits, blood pressure, lipids and triglycerides.

## Methods

### Study design

The study is a single-blinded, pragmatic RCT comparing an intervention group with a control group. The intervention group will receive individualized follow-up over 12 months, enabled through an app, while the control group receives usual care.

### Study setting and recruitment

**Fig. 1:**
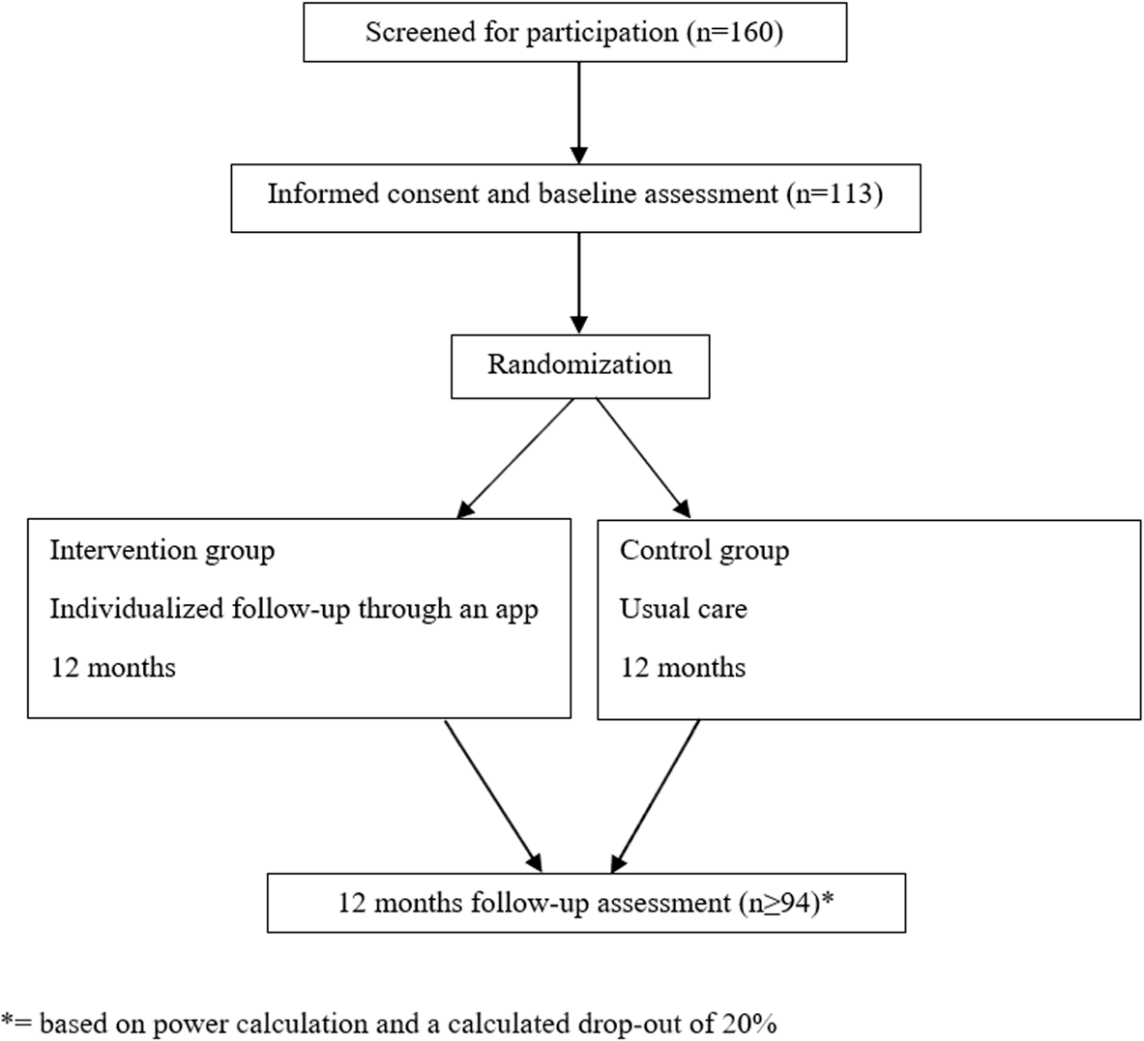
Planned flow of participants in the study

Participants will be recruited from two CR centers in the eastern part of Norway. One rehabilitation center offers a outpatient CR program for 12 weeks, while the other center offers two inpatient CR programs, one and four weeks respectively. Approximately one third of the participants will be recruited from each of the three CR programs. A researcher will visit the CR centers on a regular basis to conduct the recruitment. During CR, all patients will receive information about the study orally, as well as a request of whether they have interest in taking part in the study. If the participants are willing and able to participate in the study, they will get an appointment for baseline assessment at the time they complete the CR program. Prior to baseline assessment, the participants must provide a written informed consent. Figure 1 presents the planned flow of participants in the study.

### Inclusion and exclusion criteria

Inclusion criteria are patients who 1) have completed CR at one of the three CR programs, 2) are forty years or older, 3) are owner and user of an Android- or Apple smartphone and 4) are able to read and understand Norwegian or English. Exclusion criteria are 1) ischemia or arrhythmias uncovered at cardiopulmonary exercise test (CPET) that gives restrictions equivalent to < 80% of HR_max_ or BORG scale < 15 at exercise, 2) muscle- or skeletal disorders that affect the exercise capacity and QoL more than their heart disease and 3) severe malignant disease that affects the patient’s life span to a greater extent than their heart disease.

### Randomization

Participants will be randomly assigned at a 1:1 ratio in variable blocks to the intervention group and the control group. The randomization will be stratified to ensure equal numbers of participants from the three different CR programs in the intervention- and control group, respectively. A computer-generated, permuted block randomization scheme will be used to allocate the participants. To minimize bias, and to optimize the rigor of the RCT, a number of methodological factors have been incorporated into the design of the study. Participants will be randomly allocated to the groups via concealed allocation. Due to the nature of the intervention, it is impossible to blind the participants, nor the supervisor giving the follow-up, to the allocated groups. However, assessors for primary outcome measure will be blinded to the allocated groups. Further, intention-to-treat analyzes will be conducted to reduce bias. This maintains the random assignment to the groups and imitate the real-life situation.

### Intervention group

**Fig. 2:**
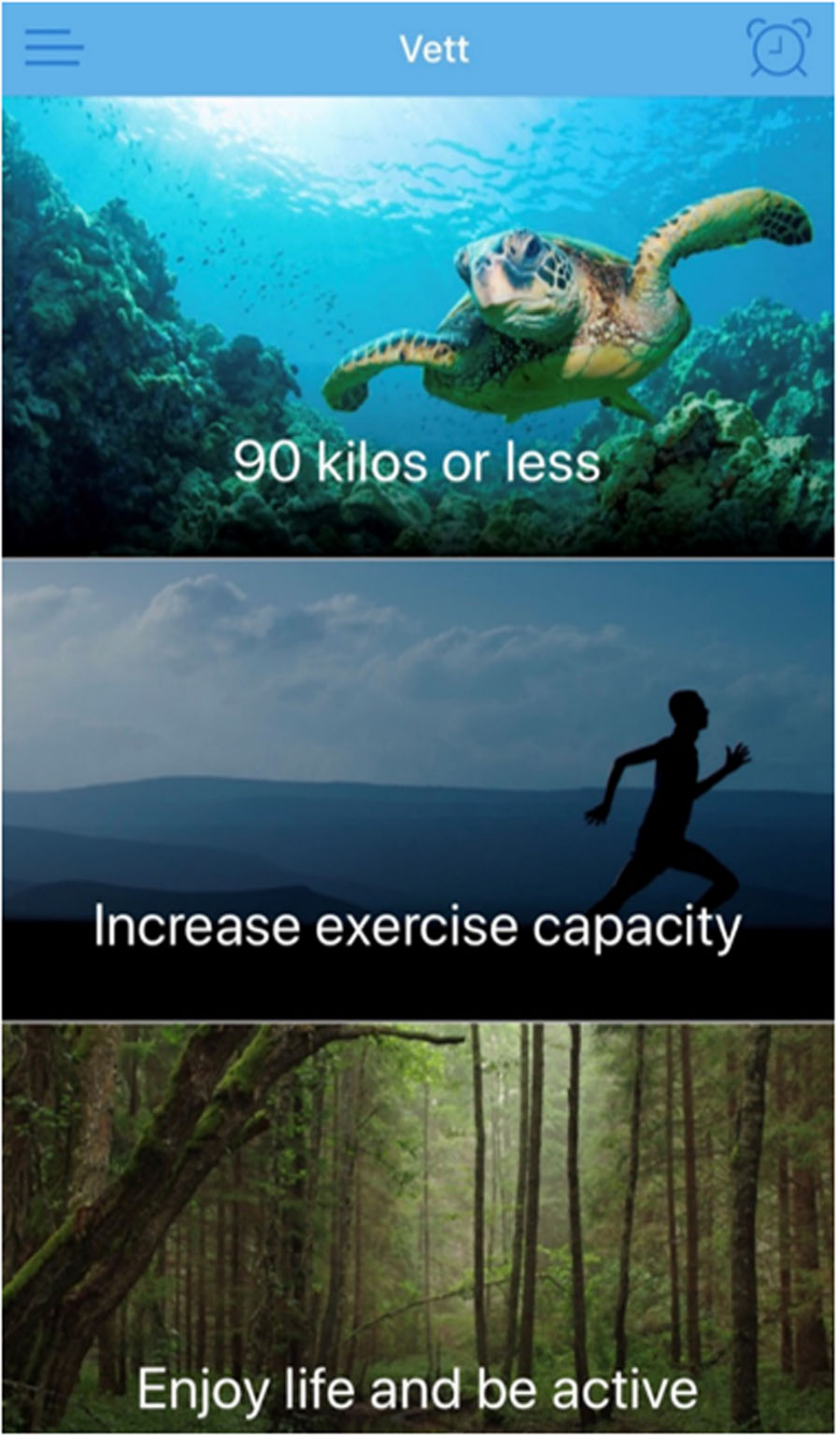
User interface of the app, showing the individual goals

**Fig. 3:**
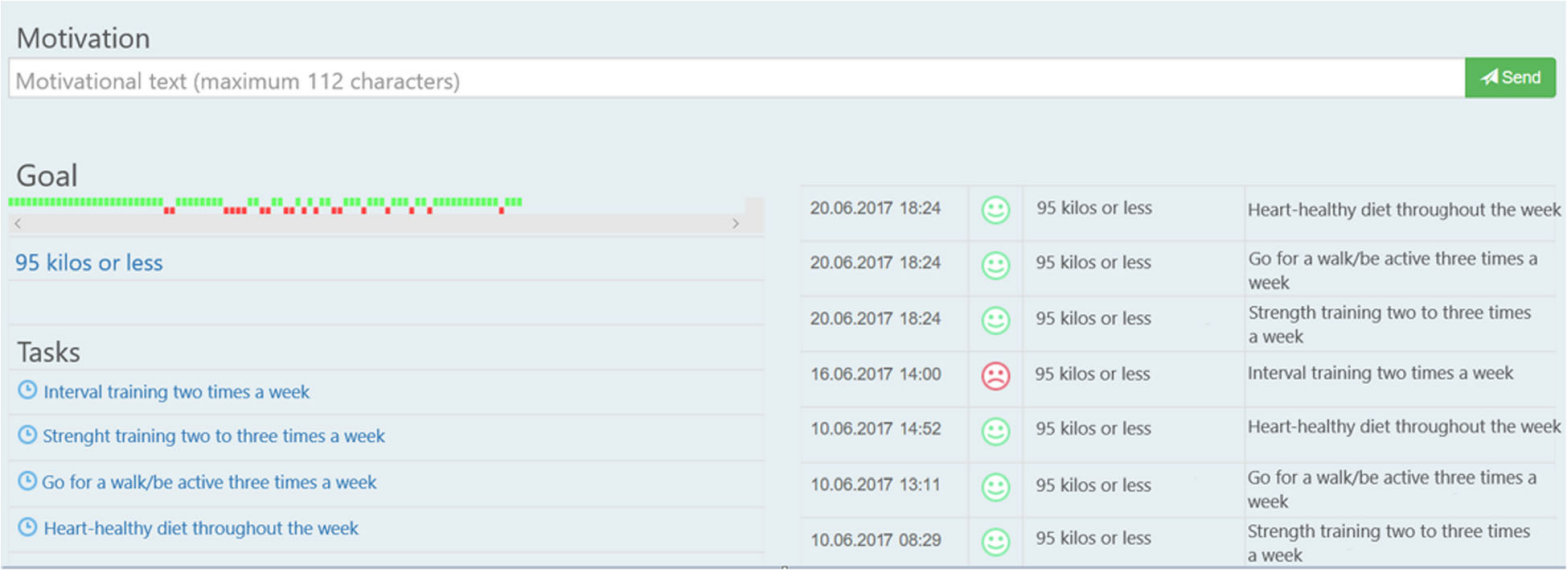
Administrator interface, showing one goal with related tasks

The intervention that will be delivered is individualized follow-up enabled by a smartphone-app. The app that will be used was developed to guide and help individuals to change behavior and/or to maintain habits. It permits the app-user to create and set goals (Fig. 2) with tasks and accompanying reminders. A supervisor has access to an administrator interface (Fig. 3) and may monitor the goals and tasks of each app-user. In addition, the app-user can write reflections in the app that the supervisor may read in the administrator interface. The app itself provides reminders and evaluations of tasks and weekly goal achievement that automatically pop up. In these evaluations, the app-user has to reply with a red or green face depending on whether they have completed the planned tasks or not, in addition to score on a Likert-scale (0–100) to evaluate the weekly goal achievement.

At baseline, a supervisor will guide each participant in setting individual goals by using elements from motivational interview. Each participant will be encouraged to set a minimum of one goal, with corresponding achievable tasks to reach the goal(s). Further, they will receive access to the app, as well as guidance in how to use it. The participant will decide when and how often reminders of the tasks should appear on their smartphone. The lower limit of amount of reminders will be one reminder each week for each task. There will not be any upper limit. During the follow-up period, the participants will receive short, tailored and individualized motivational feedback directly through the app, one to three times a week, as well as comprehensive individual feedback through e-mail once a week for the first 12 weeks, and every fourth week rest of the year. All individual feedback will be given based on what the participants have done, not done and/or their reflection notes. Participants can submit questions to the supervisor at any time, receiving an answer within two working days throughout the year. If the questions are medically related (e.g. changing medication or chest pain), they will be advised to contact their general practitioner. The participants will be monitored and followed for 12 months, by the same supervisor that includes the participants at baseline. The follow-up will be given regardless of whether the participant has used the app. In such cases, the feedback is likely to be of a more general nature.

#### Theoretical background of the intervention

The intervention in this study is based on the transtheoretical model of behavior change, also known as the stages of change model [14]. According to this model, health behavior change involves progress through six stages of change: precontemplation, contemplation, preparation, action, maintenance and termination [14]. In this connection, motivational interviews can be used to help individuals acquire motivation to change a particular behavior through collaboration, evocation, autonomy and exploration [15]. Through baseline assessment, all participants in the study will be guided in setting their own goals, that are small, important to themselves, specific and realistic to achieve [16]. The transtheoretical model takes into account that behavior change is a process over time, and that the need of support may be different in different stages [14]. The fact that the monitoring is given by a real person (the supervisor) in this study enables consideration of which stage of change each participant is in at all times. Thereby it is more likely that the feedback actually supports the participant in the change process, as the feedback may feel more apt. This is important as change through the six stages rarely occurs in a linear manner [14].

### Control group

Participants allocated to the control group will receive treatment as usual. As a part of the baseline assessment, the researcher will guide each participant in setting individual goals by using elements from motivational interview. They will be encouraged to maintain or improve their current physical activity level, exercise habits and/or a heart healthy diet, depending on their own goals and needs. They will be contacted approximately five weeks before their follow-up assessment at 12 months.

### Outcomes

The primary outcome measure will be change in VO_2peak_. Secondary outcome measures will include exercise performance, QoL, health status, health literacy, self-perceived goal achievement, exercise habits, body weight, blood pressure, lipids and triglycerides.

All assessments will be performed at baseline and at 12 months. Outcome measures will be conducted at the same CR center as the participants were attending CR. At baseline, when the participants complete their CR, demographic data, including individual goals for the next year, will be collected. The assessments will be administrated in a standardized way, which means that all assessments for each participant will be carried out in the same order.

#### Peak oxygen uptake

The participants will perform a CPET before entering the study to ensure eligibility and to measure VO_2peak._ Relative VO_2peak_ (ml/kg/min) will be the primary outcome. VO_2peak_ will be conducted and analyzed using Vyntus CPX (Vyaire Medical, Höchberg, Germany) at one of the CR centers. At the other center, Schiller Ganshorn Ergo Spirometry (Schiller AB, Baar, Switzerland) will be used. Two standardized protocols are drafted on a treadmill. The walking protocol starts at 3.5 km/h and 0% incline for 2 min, followed by increased velocity by 0.5 km/h and inclination by 1% per minute. When 6 km/h is reached, only incline increase each minute with 2%. The running-protocol starts at 5 km/h and 0% inclination for 2 min, followed by increased velocity by 1 km/h and 2% inclination respectively every other minute. The same treadmill protocol will be used at baseline and follow-up.

#### Exercise performance

Exercise performance will be evaluated as time to exhaustion, peak incline (%) and peak velocity (km/h) on the treadmill. Each minute increase in maximal treadmill time between pre- to post-test has shown to correspond with 8% decrease in risk mortality in healthy and unhealthy men [17].

#### Quality of life

Quality of life will be measured using HeartQoL. This is a health-related quality of life questionnaire and has been found to be both valid and reliable in patients referred to CR. This includes patients with angina, myocardial infarction and heart failure [18], stable coronary artery disease [19], atrial fibrillation [20], as well as patients with implantable cardioverter defibrillators [21] and patients following heart valve surgery [22].

#### Health status

Health status will be measured with EQ-5D [23]. EQ-5D consists of five questions with five answer options to each question, where a score of 1 is the best possible score and 5 is the worst possible score [23]. In addition, EQ-5D consists of an over-all health question (EQ-VAS) where the patient answers on a Likert-scale (0–100, where 0 represent the worst possible health and 100 is the best possible health) [23].

#### Health literacy

Health literacy will be measured with HLS-Q12 which is a short version of the European Health Literacy Survey Questionnaire [24]. Health literacy can be defined as an individual’s cognitive and social skills which determine motivation and ability to gain access to, understand and to use information in such a way that promote and maintain good health [25]. Health literacy is believed to have a vital impact on public health, and low health literacy has been related to unhealthy behaviors [26].

#### Self-perceived goal achievement

Self-perceived goal achievement for each goal, decided by the participant at baseline, will be assessed on a Likert-scale (0–100, where 0 represent far away from reaching the goal and 100 that the goal has been reached).

#### Exercise habits

Exercise habits will be assessed through the standardized consultation and will be defined as mean exercise sessions each week for the last year. In this context, an exercise session is defined as structured activity lasting at least 30 min where you get both sweaty and breathless, and you feel like taking a shower afterwards.

#### Body weight

Body weight will be measured prior to the CPET at both baseline and follow-up. The same equipment will be used to measure weight of the participants at pre and post-test. Participants will be weighed without shoes, wearing exercise clothes.

#### Blood pressure

The assessor of the primary outcome (blinded for group allocation) will also measure blood pressure prior to the CPET. Measurement will be done manually, preferably on the left arm. Participants will be sitting on a chair relaxing for 3–5 min before measurements will be done. Three measurements will be performed, of which the lowest measured value will be used.

#### Blood samples

Blood samples including LDL cholesterol, HDL cholesterol, total cholesterol and triglycerides will be evaluated. Participants will be encouraged to take fasting blood samples at their general practitioner at inclusion to the study and at follow-up. They are asked to bring the results to baseline and the 12 months assessment.

### Statistical procedures

The results of the study will be reported in accordance to CONSORT statement [27]. Statistical analysis will be performed using SPSS or a similar statistical package. Descriptive data will be reported for variables of interest and will be reported as mean (standard deviation), median (range) or numbers (percent) as appropriate. The data will be analyzed following the intention-to-treat principle. Prospective differences in primary and secondary outcomes and baseline characteristics between the intervention group and the control group will be assessed by t-tests for continuous and normally distributed variables and with non-parametric tests for categorical variables. To control for confounding of between group differences, multiple linear regression modelling will be used. We expect few or none missing data in baseline characteristics. However, due to possible dropouts or participants unable to complete some of the outcome measurements, there might be missing data for both primary and secondary outcome measures at follow-up. Missing data will be assessed and analyzed with appropriate methods. All tests will be two-sided. A plan for statistical analysis will be performed in advance of each paper from the study.

### Sample size

The sample size has been estimated from the primary outcome, peak oxygen consumption (VO_2peak_). A difference of 3.5 ml/kg/min in VO_2peak_ is regarded to be clinically relevant due to respectively 12–13 and 17% improvements in survival in men and women, with and without coronary heart disease [28–31]. The associated standard deviation was estimated to be 6 ml/kg/min based on the feasibility study conducted in the population [13]. This implies a moderate effect size (0.58). Given a power of 80% and level α = 0.05, a sample size of 47 in each group will be sufficient to detect a clinically relevant difference between groups. To allow for a 20% dropout, we aim at including 113 participants.

## Discussion

To our knowledge, this will be the first study to examine the effects of one-year individualized follow-up with an app after completed CR, aiming to promote adherence to lifestyle advice. We anticipate that the intervention described will have a positive impact on the primary outcome, VO_2peak_, as well as secondary outcomes such as exercise performance, body weight, QoL and self-perceived goal achievement. If the intervention proves to be effective, we will be able to support participants in the intervention group to adhere to lifestyle advice and thereby maintain the beneficial effects they have achieved in CR, which has proven to be difficult for the patient population several times [5, 32, 33]. Additionally, by evaluating the impact of the intervention, we hope to contribute to fill the knowledge gap that has been addressed by previous research in the field [3, 6, 9, 12, 34], and it is reasonable to assume that the study may be groundbreaking. Due to the large sample size and the theoretically based intervention, the study has the potential to generate new knowledge that may improve the design of future technology-based follow-up interventions of patients that have completed rehabilitation.

The intervention to be evaluated in this study can be considered as a complex intervention, and therefore we will have a systematic approach as desirable in such interventions [35]. Methodological issues may influence the validity, and thereby influence the quality of the study. In the present study, we are particularly concerned with drop-outs and participants adherence to the intervention, which may threaten internal validity. Despite that there were no drop-outs in the feasibility study [13], we are not sure whether participants will participate in the described study for the whole period or whether they show up for the 12 months assessment. Results from our feasibility study [13] demonstrated high adherence to the app. However, participants in the feasibility study only received follow-up for three months. Results from a recent systematic review on physical activity apps on healthy individuals, shows more promising results on physical activity with an intervention duration of less than three months compared to interventions lasting more than three months [36]. Participants in the intervention group who don’t use the app for four weeks will be contacted through e-mail specifically in that regard. If a response is not received within a week, we will contact the participant by telephone. Regardless of whether one succeeds in achieving contact with the participant, the follow-up will take place as described, unless the participant has given notice that he or she wishes to withdraw from the entire study. At 12 months assessment, usage of the app, e.g. percentage of answered tasks over the study period, will be extracted through the administrator interface. This enables us to review the results against actual usage of the app. To increase the probability for participants to show up at 12 months assessment, a notice will be sent out about five weeks before. In addition, we will be as flexible as possible with respect to the day of the 12 months assessment. Hopefully, this will reduce drop-outs. Nevertheless, there is a risk for drop-outs and thereby missing data. Impact of missing data will be assessed with appropriate statistical methods, e.g. linear mixed models, multiple imputations and sensitivity analysis.

By following Medical Research Council’s framework in developing the complex intervention in this study, we believe that we have done what we can to increase the quality of the study. In this respect, a clear theoretical framework is of great importance [6, 35]. Additionally, techniques such as monitoring of specific and individual goals, individual feedback and identifying barriers and developing plans for relapse prevention will be used. These are techniques which has been demonstrated as the most effective in post-CR context to increase physical activity in cardiac patients [37].

If the intervention in the described study appears effective, one of its advantages is its applicability to other diseases or health challenges where adherence to a healthy lifestyle after rehabilitation is a challenge. Therefore, if the intervention proves to be effective, a plan for implementation will be addressed.

## Data Availability

Not applicable.

## Abbreviations

CR: Cardiac rehabilitation
CV: Cardiovascular
HR: Heart rate
QoL: Quality of life
RCT: Randomized controlled trial

## References

[1] Anderson L, Thompson DR, Oldridge N, et al. Exercise-based cardiac rehabilitation for coronary heart disease. Cochrane Database Syst Rev. 2016. 10.1002/14651858.CD001800.pub3. 10.1002/14651858.CD001800.pub3 PMC6491180 26730878

[2] Myers J, McAuley P, Lavie CJ (2015). Physical activity and cardiorespiratory fitness as major markers of cardiovascular risk: their independent and interwoven importance to health status. Prog Cardiovasc Dis.

[3] Piepoli MF, Hoes AW, Agewall S (2016). 2016 European guidelines on cardiovascular disease prevention in clinical practice: the sixth joint task force of the European Society of Cardiology and Other Societies on cardiovascular disease prevention in clinical practice (constituted by representatives of 10 societies and by invited experts) developed with the special contribution of the European Association for Cardiovascular Prevention & rehabilitation (EACPR). Atherosclerosis.

[4] Letnes JM, Dalen H, Vesterbekkmo EK, et al. Peak oxygen uptake and incident coronary heart disease in a healthy population: the HUNT fitness study. Eur Heart J. 2018. 10.1093/eurheartj/ehy708. 10.1093/eurheartj/ehy708 30496487

[5] Pinto BM, Goldstein MG, Papandonatos GD (2011). Maintenance of exercise after phase II cardiac rehabilitation. Am J Prev Med.

[6] Beatty AL, Fukuoka Y, Whooley MA. Using mobile technology for cardiac rehabilitation: a review and framework for development and evaluation. J Am Heart Assoc. 2013;2:e000568 Research Support, N.I.H., Extramural Review. 10.1161/JAHA.113.000568. 10.1161/JAHA.113.000568 PMC3886753 24185949

[7] Gandhi S, Chen S, Hong L (2017). Effect of mobile health interventions on the secondary prevention of cardiovascular disease: systematic review and meta-analysis. Can J Cardiol.

[8] Marzano L, Bardill A, Fields B (2015). The application of mHealth to mental health: opportunities and challenges. Lancet Psychiatry.

[9] Widmer RJ, Allison TG, Lerman LO (2015). Digital health intervention as an adjunct to cardiac rehabilitation reduces cardiovascular risk factors and rehospitalizations. J Cardiovasc Transl Res.

[10] Maddison R, Rawstorn JC, Stewart RAH (2019). Effects and costs of real-time cardiac telerehabilitation: randomised controlled non-inferiority trial. Heart.

[11] Varnfield M, Karunanithi M, Lee CK (2014). Smartphone-based home care model improved use of cardiac rehabilitation in postmyocardial infarction patients: results from a randomised controlled trial [with consumer summary]. Heart 2014 Nov 15.

[12] Forman DE, LaFond K, Panch T, et al. Utility and efficacy of a smartphone application to enhance the learning and behavior goals of traditional cardiac rehabilitation: a feasibility study. J Mol Signal. 2014;34:327–34 Observational Study. 10.1097/HCR.0000000000000058. 10.1097/HCR.0000000000000058 24866355

[13] Lunde P, Nilsson BB, Bergland A (2019). Feasibility of a Mobile Phone App to Promote Adherence to a Heart-Healthy Lifestyle: Single-Arm Study. JMIR Form Res.

[14] Prochaska JO, Redding CA, Evers KE. The transtheoretical model and stages of change. In: Glanz K, Rimer BK, Viswanath K, editors. Health behavior: Theory, research and practice: Wiley-Blackwell; 2015. p. 125–48.

[15] Rollnick S, Miller W. Motivational interviewing: preparing people to change addictive behaviour. N Y. 1991.

[16] Miller WR, Rollnick S. Preparing people for change. Motiv Int. 2002.

[17] Blair SN, Kohl HW, Barlow CE (1995). Changes in physical fitness and all-cause mortality: a prospective study of healthy and unhealthy men. Jama.

[18] Oldridge N, Höfer S, McGee H (2014). The HeartQoL: part II. Validation of a new core health-related quality of life questionnaire for patients with ischemic heart disease. Eur J Prev Cardiol.

[19] De Smedt D, Clays E, Höfer S (2016). Validity and reliability of the HeartQoL questionnaire in a large sample of stable coronary patients: the EUROASPIRE IV study of the European Society of Cardiology. Eur J Prev Cardiol.

[20] Kristensen MS, Zwisler A-D, Berg SK (2016). Validating the HeartQoL questionnaire in patients with atrial fibrillation. Eur J Prev Cardiol.

[21] Zangger G, Zwisler A-D, Kikkenborg Berg S (2018). Psychometric properties of HeartQoL, a core heart disease-specific health-related quality of life questionnaire, in Danish implantable cardioverter defibrillator recipients. Eur J Prev Cardiol.

[22] Grønset CN, Thygesen LC, Berg SK, et al. Measuring HRQoL following heart valve surgery: the HeartQoL questionnaire is a valid and reliable core heart disease instrument. Qual Life Res. 2019:1–9. 10.1007/s11136-018-02098-1 30610503

[23] Herdman M, Gudex C, Lloyd A (2011). Development and preliminary testing of the new five-level version of EQ-5D (EQ-5D-5L). Qual Life Res.

[24] Finbråten HS, Wilde-Larsson B, Nordström G (2018). Establishing the HLS-Q12 short version of the European health literacy survey questionnaire: latent trait analyses applying Rasch modelling and confirmatory factor analysis. BMC Health Serv Res.

[25] Nutbeam D (2000). Health literacy as a public health goal: a challenge for contemporary health education and communication strategies into the 21st century. Health Promot Int.

[26] Kickbusch I PJ, Apfel F, Tsouros AD (eds). Health Literacy. The solid facts. http://www.euro.who.int/__data/assets/pdf_file/0008/190655/e96854.pdf (2013, accessed 21 May 2019).

[27] CONSORT Statement 2010, http://www.consort-statement.org/consort-2010 (accessed 7 May 2019).

[28] Myers J, Prakash M, Froelicher V (2002). Exercise capacity and mortality among men referred for exercise testing. N Engl J Med.

[29] Kokkinos P, Myers J, Kokkinos JP (2008). Exercise capacity and mortality in black and white men. Circulation.

[30] Gulati M, Pandey DK, Arnsdorf MF (2003). Exercise capacity and the risk of death in women: the St James women take heart project. Circulation.

[31] Valkeinen H, Aaltonen S, Kujala U (2010). Effects of exercise training on oxygen uptake in coronary heart disease: a systematic review and meta-analysis. Scand J Med Sci Sports.

[32] Willich S, Müller-Nordhorn J, Kulig M (2001). Cardiac risk factors, medication, and recurrent clinical events after acute coronary disease. A prospective cohort study. Eur Heart J.

[33] Kotseva K, Wood D, Backer GD (2009). EUROASPIRE III: a survey on the lifestyle, risk factors and use of cardioprotective drug therapies in coronary patients from 22 European countries. Eur J Cardiovasc Prev Rehabil.

[34] Lunde P, Nilsson BB, Bergland A (2018). The effectiveness of smartphone apps for lifestyle improvement in noncommunicable diseases: systematic review and meta-analyses. J Med Internet Res.

[35] Craig P, Dieppe P, Macintyre S (2013). Developing and evaluating complex interventions: the new Medical Research Council guidance. Int J Nurs Stud.

[36] Romeo A, Edney S, Plotnikoff R (2019). Can smartphone apps increase physical activity? Systematic Review and Meta-Analysis. J Med Internet Res.

[37] Ferrier S, Blanchard CM, Vallis M (2011). Behavioural interventions to increase the physical activity of cardiac patients: a review. Eur J Cardiovasc Prev Rehabil.

